# Temporal Dynamics of Disgust and Morality: An Event-Related Potential Study

**DOI:** 10.1371/journal.pone.0065094

**Published:** 2013-05-28

**Authors:** Qun Yang, Li Yan, Junlong Luo, An Li, Ye Zhang, Xuehong Tian, Dexuan Zhang

**Affiliations:** 1 College of Education Sciences, Hangzhou Normal University, Hangzhou, P. R. China; 2 College of Education, Shanghai Normal University, Shanghai, P. R. China; 3 School of Law, Hangzhou Normal University, Hangzhou, P. R. China; 4 Center for Cognition and Brain Disorder, Hangzhou Normal University, Hangzhou, P. R. China; University of Nottingham Malaysia Campus, Malaysia

## Abstract

Disgust is argued to be an emotion that motivates the avoidance of disease-causing entities in the physical domain and unacceptable behaviors in the social-moral domain. Empirical work from behavioral, physiological and brain imaging studies suggests moral judgments are strongly modulated by disgust feelings. Yet, it remains unclear how they are related in the time course of neural processing. Examining the temporal order of disgust emotion and morality could help to clarify the role of disgust in moral judgments. In the present research, a Go/No-Go paradigm was employed to evoke lateralized readiness potentials (LRPs) to investigate the temporal order of physical disgust and moral information processing. Participants were asked to give a “yes” or “no” response regarding the physical disgust and moral wrongness of a social act. The results showed that the evaluation of moral information was processed prior to that of physical disgust information. This suggests that moral information is available earlier than physical disgust, and provides more data on the biological heterogeneity between disgust and morality in terms of the time course of neural activity. The findings implicate that physical disgust emotion may not be necessary for people to make moral judgments. They also suggest that some of our moral experience may be more fundamental (than physical disgust experience) to our survival and development, as humans spend a considerable amount of time engaging in social interaction.

## Introduction

Disgust is regarded as one of the basic emotions, as it meets critical criteria that are considered to be essential to any basic emotion [1]. Disgust emotion is typically marked by behavioral withdrawal from some objects and, in terms of facial expression, it is reflected in the retraction of the upper lip and wrinkling of the nose. The experience of disgust leads to a strong feeling of revulsion which can be associated with specific physiological reactions, such as nausea and vomiting [1], [2]. The output system of disgust has remained constant during human evolution. However, according to Rozin et al.(2000), the elicitors of disgust have expanded from the physical domain, such as contaminated food, human and animal waste, poor hygiene, contact with dead bodies, and so on, to the social-moral domain, such as interpersonal disgust, improper sexual behaviors and moral violations [1]. Disgust elicitors in the physical domain are mostly concrete stimuli and are related to contamination that may cause physical diseases in the human body. Moral disgust, on the other hand, is more abstract and is related to a variety of moral violations which could be a threat to the “human soul”. The function of disgust is therefore claimed to have been transformed from “the guardian of the mouth” to “the guardian of the soul” [1], [3], [4].

The physical and social aspects of disgust have long been recognized. For instance, Darwin (1872/1965, p.253) referred to disgust as “something revolting, primarily in relation to the sense of taste, and to anything which causes a similar feeling”[5]. Rozin et al. (2000) proposed a process parallel to the concept of preadaptation in evolutionary biology in order to explain the mechanism underlying the expansion of disgust [1]. Just as the human mouth has evolved to speak and is not merely restricted to basic functions such as eating, so the disgust system has evolved; in addition to the avoidance of disease-causing substances, the disgust system now functions to reject moral violations.

Studies have shown that people in different cultures not only commonly experience revulsion towards contaminated food and animal waste, but also feel disgust in response to many immoral acts [3], [6], [7]. When participants experienced moral threats in a task in which they were reminded of moral violations from their own experience or that of another person, the mental accessibility of cleansing-related words was found to increase, as was the psychological desire for cleansing [8]. Chapman et al. (2009) found that disgust in response to unfair treatment in an economic game activated the same facial muscle as disgust elicited by disease vectors and bad tastes. The authors claimed that moral disgust may have evolved from more primitive forms of disgust related to distaste and contamination [9].

Evidence has so far been obtained to support that the two types of disgust experience evoke similar mental feelings, share the same facial expression, and may even have partially overlapping physiological and neural components [1]–[3], [9]–[13]. However, our compulsion to avoid objects such as feces and bad food may be fundamentally different from feelings of disgust elicited by immoral actions like stealing and killing. Simpson et al. (2006) demonstrated that picture cues of the two types of disgust produced different emotional responses, and the intensity of the response changed differently over time. In addition, a gender-based difference was apparent, but only for the responses to physical disgust elicitors [14]. Evidence from neuroimaging research has shown that the neural networks activated by basic disgust and moral disgust scenarios covered distinct brain regions [10]–[13].

Knowledge of common and different mechanisms underlying disgust and morality would enhance our understanding regarding the nature of disgust and moral judgments. To the best of our knowledge, few studies have so far been carried out to investigate the relationship between disgust and morality in terms of temporal dynamics. Accordingly, the first goal of the present research is to investigate how feelings of disgust and a sense of morality differ in the time course of neural processing using event-related potentials (ERPs).

Disgust is considered as one of the most prototypical moral emotions and it has been found that the manipulation of physical disgust emotion can influence people's moral judgments [15], [16]. Judgments of moral violations were harsher when associated with different disgust experience [17], [18]. This effect was shown to be reversed when disgust feelings were reduced using a cleanliness manipulation [19]. Although most evidence has shown that disgust emotion simply amplifies moral judgments, a couple of notable studies seem to suggest that disgust is sufficient to instigate negative moral judgments. For example, previous research found that morally neutral acts were judged morally worse when participants felt disgust in a hypnotic procedure or when watching a disgusting film clip [20], [21]. Based on the current evidence, Huebner et al. (2009) suggest that we cannot draw any definite conclusions regarding whether our intuitive moral judgments are driven by emotional processes. They further proposed that evidence from time-locked functional magnetic resonance imaging (fMRI) and ERP studies was necessary in order to elucidate the effect of emotion on moral judgments [22].

Sarlo et al. (2011) carried out a pioneering study using ERPs in order to examine the interaction between emotion and cognition in moral decision making. They found that the more aversive moral choice evoked a larger early negative component that peaked around 260 ms, while the choice requiring more cognitive control induced greater later positive potentials [23]. As noted by the researchers themselves, the electroencephalogram (EEG) data were time-locked at the point when participants had to decide between two choices after reading a scenario. Each option was such an integral part of a moral dilemma that it was hard to isolate the processing phases. Furthermore, little is known about the questions of whether there is a temporal order of disgust and morality and when disgust emotion is extracted from moral judgments.

Thus, the second goal of this research is to reveal whether there is a temporal order of disgust and morality, and furthermore, to understand when disgust emotion is extracted from moral judgments. If disgust feelings are necessary to causally lead to moral judgments, we would expect that people should feel disgust emotion before they make a judgment of what is right or wrong, as a cause precedes its effects in a causal relationship [24].

The Go/No-Go paradigm of LRPs is typically used to help characterize the temporal order of information extraction by comparing LRPs in the Go vs. No-Go conditions. An LRP is an ERP that is elicited when a participant initiates a movement with hands or feet. It displays maximal amplitudes at scalp sites over the motor cortex contralateral to the moving part of the body and reflects the preparation of motor activity [25]–[27]. When a Go/No-Go paradigm is applied, subjects are asked to respond with their left or right hand based on a certain attribute of a presented target on Go trials. For example, they may be asked to respond to the letter S with the left hand and T with the right hand. For No-Go trails, participants are asked to withhold their hand selection responses according to some other feature of the target. For example, they may be asked not to respond if the letter has a small size. An LRP on No-Go trials would indicate that the processing of the feature that is driving the hands selection was processed prior to that of the feature that informs the participant not to respond. Miller & Hackley (1992) reported an apparent LRP on No-Go trials when the shape information of a letter (S or T) cued the hand selection and the size information (S or s) determined whether a response should be withheld, which indicated that the easily discriminated shape feature initiated a response even before the size analysis signified that no response was necessary [27].

In the current study, the Go/No-Go paradigm of LRP component was employed in order to examine whether disgust information is extracted before or after moral information. Participants were asked to judge the nature of a social action in terms of physical disgust and moral wrongness. The features of physical disgust and morality of an act were mapped onto Go or No-Go trials. Each participant was tested in two experimental sessions. In the first session, the feature of morality of an act determined the hand selection response (left/right) and the feature of physical disgust indicated whether a response should be withheld. For example, in one of the configurations, participants were told to respond with their left hand if an act was morally wrong and to respond with their right hand if an act was morally acceptable. They were required to respond only when physical disgust was felt. The features that were mapped to hand selection and the Go/No-Go instruction were swapped in the second session. By comparing LRPs in the Go vs. No-Go conditions, we aimed to characterize the temporal order of physical disgust and morality processing.

We hypothesized that if the two types of information (morality and physical disgust) are accessed at different points during the judgment of a social action, a No-Go LRP should be evident in one of the aforementioned sessions. In other words, the feature that determines the selection of a left or right response hand would be available for processing before the other feature which informs the participant whether to respond or not.

## Methods

### Participants

Sixteen healthy undergraduate and graduate students of Hangzhou Normal University (10 females), aged 24±3 years, took part in the experiment. The study was approved by the local ethics committee of Hangzhou Normal University. Each volunteer signed a consent form before the experiment and was paid 25 RMB for their participation. All participants reported being right-handed with normal or corrected-to-normal vision. Following instruction and prior practice, all participants were able to perform the task successfully.

### Stimuli and procedure

The study comprised 224 stimuli and included four types of short statements (presented in Table 1) depicting acts that are: morally wrong and physically repulsive (WD: wrong and disgusting); morally wrong but devoid of physical disgust elicitors (WN: wrong and non-disgusting); morally neutral but physically aversive (ND: neutral and disgusting) and morally neutral and emotionally neutral (NN: neutral and non-disgusting). To ensure that the stimuli fell into the four categories as expected, an independent sample of 59 people was recruited to evaluate each statement in terms of morality and physical disgust. These individuals were asked questions such as “do you think the act of a person drinking (human) blood at a party is immoral” and “does the fact that a person is drinking (human) blood make you feel nauseated”.

**Table 1:** Examples of the statements used and responses made in session 1 of the Go/No-Go paradigm.

	Left hand	Right hand
**Go**	A person at a party is drinking blood (WD)	A person at a party is drinking urine (ND)
**No-Go**	A person at a party is stealing money (WN)	A person at a party is drinking water (NN)

For the first experimental session, the physical disgust feature of an act (disgusting or non-disgusting) determined whether or not a response was to be made while the feature of morality (wrong or right) determined whether to respond using the left (pressing the “A” key) or the right hand (pressing the “L” key). For example, participants responded to a morally wrong act with the left hand and to a morally acceptable act with the right hand. A response was executed when the act was physically disgusting, but was withheld when the act was not disgusting (see Table 1). In the second session, the procedure was similar except that the feature of morality determined the Go and No-Go response while the feature of physical disgust determined the response hand.

**Figure 1:**
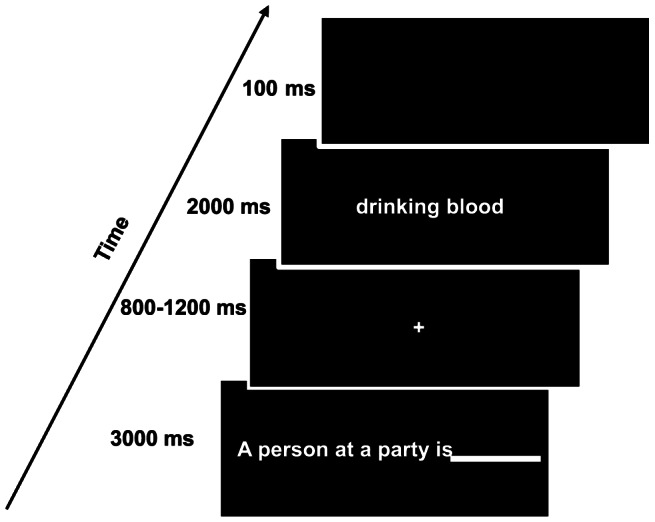
An illustration of the sequence of events in an experimental trial.

Figure 1 shows the sequence of events in a trial for the two sessions. It was considered that the presentation of a long sentence may cause eye movements and thus contaminate the EEG signal. Therefore, each statement was separated into two parts by a screen: the scene words part (for example, “a person at a party is”) was presented before the key words part (“drinking blood”). Participants were asked to respond upon finishing reading the second part of a statement, the presentation of which was terminated by the key pressing or after 2000 ms. The features of the key words parts (e.g. the number of words) were carefully matched across the four types of acts.

LRP data recording was time-locked to the onset of the key words of a statement. The left/right hand response and Go/No-Go assignments, and the order of the two experimental sessions were counterbalanced across subjects.

After the experiment, participants were asked to make a judgment for each statement according to the morality and physical disgust by responding “yes” or “no” to each question. Those statements to which participants responded inconsistently during and after the experiment were eliminated from the data analysis.

### Electrophysiological recording and analysis

EEG was recorded during key words presentation using a 64-channel BrainCap (Brain Products GmbH, Munich, Germany). All electrode sites were referenced online to FCz and re-referenced offline to the average of the left and right mastoids. Vertical electrooculogram (EOG) was recorded supra-orbitally at the left eye. Horizontal EOG was recorded from the right orbital rim. The EEG and EOG signals were amplified using a DC 1000 Hz bandpass and were continuously sampled at 500 Hz/channel. All inter-electrode impedance was maintained below 10 kΩ.

EEG data were analyzed using Brain Vision Analyzer (version 2.0). The averaging of ERPs was computed offline. Eye movement artifacts (blinks and eye movements) were rejected offline using the Gratton and Coles algorithm [28]. All single-trial waveforms containing movement artifacts in the time window from 200 ms before target onset to 2000 ms after target onset were removed from the data. The data were filtered offline using a 0.01–30.00 Hz bandpass infinite impulse response (IIR) filter. The number of valid trials for the analysis was no less than 40 for each type of the statements (WD, WN, ND and NN) in the two experimental sessions.

LRPs are classically recorded from the electrodes on the left (C3) and right (C4) side of the scalp over the motor cortex, and are associated with larger negativity contralateral to the moving part of the body [29]. The average ERPs for the left hand movement were calculated by subtracting the signal of C3 from C4, and for the right hand movement by subtracting the C4 signal from C3. These differences for both the left and right side were averaged as the final LRP (LRP = [left hand (C4−C3) + right hand (C3−C4)]/2) [30]–[32]. The LRP is characterized by a negative-going deflection which is observed as soon as response preparation for the cued response hand occurs.

To define the presence of an LRP and to determine its onset, we conducted a moving time window analysis with 50-ms intervals, starting from the onset and continuing in sequential steps of 10 ms (for example, 690–740 ms, 700–750 ms and so on). For each time window, we applied a two-tailed t test to determine whether the mean voltage of a window was significantly higher than that of the baseline. The onset of the first of five consecutive windows in which the mean voltage of each LRP had a significant t value was used to determine LRP onset latency [33].

## Results

### Ratings of moral wrongness and physical disgust

**Figure 2:**
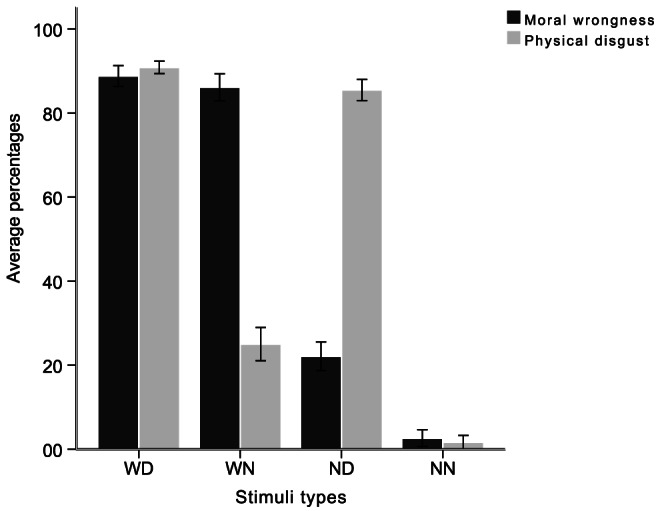
The average percentages of “immoral” and “disgusting” responses for each type of the stimuli. WD = wrong and disgusting; WN = wrong and non-disgusting; ND = (morally) neutral and disgusting; NN = (morally) neutral and non-disgusting. Error bars: 95% CI.

Prior to the ERP experiment, we collected the ratings of moral wrongness and physical disgust for each statement. The percentage of participants who considered a statement to be immoral or physically disgusting was calculated; the average percentages for each stimuli type are indicated in Figure 2. An analysis of variance (ANOVA) conducted on the proportion of “immoral” responses for moral judgment, with the type of statements as a within-subject factor, revealed a significant effect (F (3, 165) = 862.69, p<0.001, η_p_^2^ = 0.94). As seen in Figure 2, the percentage of participants who viewed a statement as morally wrong was much higher in WD and WN statements than in ND and NN statements. An ANOVA carried out on the proportion of “disgusting” responses for disgust judgment, with the type of statements as a within-subject factor, also yielded a significant effect (F (3, 165) = 1172.49, p<0.001, η_p_^2^ = 0.96). The majority of participants reported feeling physically disgusted by WD and ND statements, while only a small number had the same feeling for WN and NN statements. It is interesting to note that more participants judged ND statements as morally wrong and WN statements as disgusting compared with judgments of NN statements (both Bonferroni pairwise comparisons were significant, p<0.001). Overall, the basic behavioral results were consistent with our assumptions regarding the classification of the materials, which meant that there were enough trials in each condition for the LRP analysis.

### LRP data

A statistical analysis of reaction time data revealed no significant differences between left and right hand responses in the two sessions (F (1,15) = 1.022, p = 0.328, η_p_^2^ = 0.064). The main aim of the study was to examine the temporal order of moral and physical disgust information processing by comparing LRPs using the Go/No-Go paradigm.

**Figure 3:**
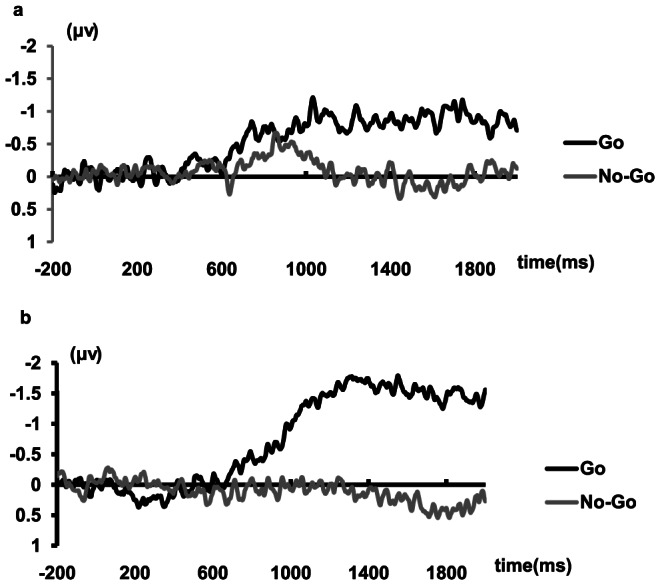
Grand average LRPs on Go trials and No-Go trials. (a) Session 1. (b) Session 2.

In the first session, as shown in Figure 3a, significant LRPs were observed from 690 ms after the onset of the key words for Go trials (Two-tailed t test results of LRPs for the five consecutive windows starting from 690 ms were: t = −2.373, p = 0.031; t = −3.345, p = 0.004; t = −2.727, p = 0.016; t = −3.363, p = 0.004; and t = −2.457, p = 0.027, respectively.). For No-Go trials, the onset time of LRPs was 800 ms (Two-tailed t test results of LRPs for the five consecutive windows starting from 800 ms were: t = −2.193, p = 0.044; t = −2.510, p = 0.024; t = −2.109, p = 0.052; t = −2.174, p = 0.046; and t = −2.131, p = 0.050, respectively.). However, no significant difference of LRPs was found between Go and No-Go trials during the 690–800 ms interval (t = −0.906, p = 0.379). In the 800−1030 ms time window, the Go and No-Go LRPs were remarkably different from the baseline (t = −2.365, p = 0.032; t = −2.587, p = 0.040), yet did not differ from each other (t = −577, p = 0.573). After 1030 ms, an LRP was only present on Go trials, having disappeared on No-Go trials.

To exclude the possibility that the effect in the first session was caused by the task configuration (the response hand selection preceded the Go/No-Go decision), in the second session, participants were asked to select a response hand based on the physical disgust feature, and to make the Go/No-Go decision based on the feature of morality. As seen in Figure 3b, significant LRPs were only observed on Go trails from 860 ms after the onset of the key words (t = −2.161, p = 0.047; t = −2.242, p = 0.041; t = −2.251, p = 0.040; t = −2.258, p = 0.039; t = −2.2, p = 0.044), while there was no significant LRP for No-Go trials.

## Discussion

This study was designed to investigate the temporal features of disgust and morality by using the Go/No-Go paradigm to evoke LRP components to address the temporal order of information extraction processing. The results revealed a negative-going wave between 800 ms and 1030 ms for both Go and No-Go trials in the first session. The presence of a short period of LRPs for No-Go trials indicates that the availability of moral information enabled the preparation of the response hand selection. However, an overt response was not carried out as participants immediately realized the presence of the physical disgust feature. Importantly, no LRP was observed on No-Go trials in the second session. This confirms that the effect was not associated with the fact that the left/right hand decision was made earlier than the Go/No-Go response.

When the LRP is considered as a physiological index, the findings indicate that the feature of morality of an act initiated an unconscious impulse of motor responses for hand selection prior to the conscious realization that responses were to be inhibited. This study revealed a number of important findings regarding the nature of the relationship between disgust and morality and the role of disgust in moral judgments.

Firstly, our results showed that the processing of physical disgust and moral information occurred at different phases. This further supports the claim that the avoidance system for concrete disgust elicitors, such as feces, is neurally different in terms of the time course of processing from that of some harmful social behaviors, such as theft [10], [12], [13]. The fact that physical disgust and moral intuition may be essentially different suggests that we may need different assessments to measure disgust experience in the physical and social-moral domain. In the development of a scale that measures the disgust sensitivity of individuals, items in the social-moral domain were found to be only weakly correlated with those in other domains [34], [35].

More importantly, the examination of the discrete mechanisms underlying the two types of disgust experience (physical and moral disgust) could improve the diagnosis and treatment of clinical populations with impairments in disgust emotion, such as obsessive-compulsive disorders and psychopathy patients, and different types of law offenders. In a study of Huntington's disease patients, Hayers et al. (2007) found that the impairment of disgust processing was restricted solely to physical disgust while the ability to detect moral disgust remained intact [36]. It would be interesting to know whether criminals with aggressive behaviors show a similar pattern of results to these patients.

Secondly, the temporal priority of morality over physical disgust further suggests that the physical disgust emotion may not be necessary for people to make moral judgments. Some recent research seems to claim that disgust emotion is necessary for moral judgments [3], [18], [37] and that impairments in disgust may explain some psychopathic behaviors [38]. Yet in spite of the bulk of empirical work implying that disgust plays a central role in human moral judgments, it remains unclear whether they are causally connected [39]. Our results suggest that people can unconsciously respond to moral information faster than pure disgust; therefore, it may not be necessary for physical disgust to be present in order for people to determine the moral wrongness of a behavior.

Finally, our results suggest people could unconsciously respond to moral information faster than pure disgust; therefore it may be more fundamental than physical disgust to the social development of human beings. There is a longstanding debate as to whether moral judgment is the product of conscious reasoning or intuitive responses [40], [41]. In recent decades, the dominance of rationalism in moral psychology has gradually shifted to intuitive primacy. One of the most influential theories featuring moral intuitions is the social intuitionist model [42]. According to the model, moral judgment is a process of fast, automatic intuition similar to aesthetic judgments and perception. Deliberative systems only serve to justify the results of a moral judgment that has already been made. Conscious reasoning has minimal influence on our moral judgments unless a judgment is reached via rigid reasoning procedures [3], [42]. As evidenced by the so-called moral dumbfounding effect, it has been found that individuals can make moral judgments without being able to articulate adequate reasons [43]. For example, many people would regard consensual incest between siblings as wrong though they cannot give specific reasons. In a web-based survey, Cushman et al. (2006) found that individuals had great difficulty adequately justifying the patterns of judgments that conformed to the moral principle that intentional harm is worse than unintentional harm. Thus, they favored the claims that, at least in some situations, individuals use their intuition instead of explicit moral rules to reach a judgment [44].

The faster availability of moral intuition in the current study may suggest that moral experience is fundamental to human survival and development. Physical disgust helps the human body to avoid touching toxic and disease-causing objects in the physical environment, whereas moral disgust helps the human mind to distinguish between right and wrong in the social environment so that individuals can keep their distance from transgressors or the thought of committing a transgression. Harsh though nature might be and as primal as feelings of physical disgust are, human beings now interact more with the social environment; threats posed by the social environment are much more common and complex than those generated by the physical environment as they develop phylogenetically and ontogenetically.

In conclusion, we isolated the processing phases of physical disgust and morality in individuals judging a social act by applying the Go/No-Go paradigm to evoke LRP components. The temporal priority of morality over physical disgust in this study may shed some light on the nature of the two types of experience. Moreover, it helps to elucidate the role of disgust emotion in moral judgments. However, further research is needed to provide more data on the time course of neural activity associated with disgust and moral judgments. This research should attempt to address the limitations of the current study. For instance, the stimuli used in this study were written statements. Language may give moral information some advantages in the time course of processing. Although semantic stimuli have been used effectively to evoke physical disgust in previous research [10], [11], [13], it is possible that the extraction of disgust information may have been delayed due to the abstract format of the stimuli. However, it was hard to find images in a visually vivid format that reflected all the moral behaviors in the present study and it would be difficult to match the irrelevant features of the pictorial stimuli across the four conditions. Therefore, we cannot exclude the possibility that the temporal priority of moral disgust over physical disgust might be limited to verbally presented information.
